# Induction of Stable Drug Resistance in Human Breast Cancer Cells Using a Combinatorial Zinc Finger Transcription Factor Library

**DOI:** 10.1371/journal.pone.0021112

**Published:** 2011-07-19

**Authors:** Jeongeun Lee, Andrew S. Hirsh, Ben S. Wittner, Morgan L. Maeder, Rajasekhar Singavarapu, Magdalena Lang, Sailajah Janarthanan, Ultan McDermott, Vijay Yajnik, Sridhar Ramaswamy, J. Keith Joung, Dennis C. Sgroi

**Affiliations:** 1 Molecular Pathology Unit, Massachusetts General Hospital, Charlestown, Massachusetts, United States of America; 2 Center for Cancer Research, Massachusetts General Hospital, Charlestown, Massachusetts, United States of America; 3 Center for Computational and Integrative Biology, Massachusetts General Hospital, Boston, Massachusetts, United States of America; 4 Department of Pathology, Harvard Medical School, Boston, Massachusetts, United States of America; 5 Department of Medicine, Massachusetts General Hospital, Boston, Massachusetts, United States of America; 6 Gastrointestinal Unit, Massachusetts General Hospital, Boston, Massachusetts, United States of America; 7 Wellcome Trust Sanger Institute, Genome Research Limited, Hinxton, United Kingdom; University of Texas Southwestern Medical Center at Dallas, United States of America

## Abstract

Combinatorial libraries of artificial zinc-finger transcription factors (ZF-TFs) provide a robust tool for inducing and understanding various functional components of the cancer phenotype. Herein, we utilized combinatorial ZF-TF library technology to better understand how breast cancer cells acquire resistance to fulvestrant, a clinically important anti-endocrine therapeutic agent. From a diverse collection of nearly 400,000 different ZF-TFs, we isolated six ZF-TF library members capable of inducing stable, long-term anti-endocrine drug-resistance in two independent estrogen receptor-positive breast cancer cell lines. Comparative gene expression profile analysis of the six different ZF-TF-transduced breast cancer cell lines revealed five distinct clusters of differentially expressed genes. One cluster was shared among all 6 ZF-TF-transduced cell lines and therefore constituted a common fulvestrant-resistant gene expression signature. Pathway enrichment-analysis of this common fulvestrant resistant signature also revealed significant overlap with gene sets associated with an estrogen receptor-negative-like state and with gene sets associated with drug resistance to different classes of breast cancer anti-endocrine therapeutic agents. Enrichment-analysis of the four remaining unique gene clusters revealed overlap with myb-regulated genes. Finally, we also demonstrated that the common fulvestrant-resistant signature is associated with poor prognosis by interrogating five independent, publicly available human breast cancer gene expression datasets. Our results demonstrate that artificial ZF-TF libraries can be used successfully to induce stable drug-resistance in human cancer cell lines and to identify a gene expression signature that is associated with a clinically relevant drug-resistance phenotype.

## Introduction

Combinatorial libraries of artificial zinc-finger transcription factors (ZF-TFs) provide a powerful tool for inducing and understanding important cellular phenotypes [1], [2]. Zinc fingers are compact ∼30 amino acid domains that can be engineered to bind various three bp DNA “subsites” [3], [4]. By “mixing and matching” collections of individual zinc fingers with various pre-selected DNA-binding specificities, large collections (or “libraries”) of multi-finger arrays, each predicted to bind a different spectrum of target DNA sequences, can be easily assembled [1], [2]. These multi-finger arrays can in turn be fused to transcriptional regulatory domains to create libraries of artificial ZF-TFs capable of activating or repressing expression of specific genes. Previous studies have shown that such libraries can be screened to identify specific ZF-TFs capable of inducing phenotypes of interest in bacteria, yeast, and mammalian cells [1], [2], [5], [6], [7], [8], [9], [10], [11], [12].

We sought to use combinatorial ZF-TF library technology to induce resistance to fulvestrant, a clinically important anti-endocrine therapeutic agent. In the United States, approximately 70% of all breast cancer patients are diagnosed with estrogen receptor (ER)-positive breast cancer and anti-endocrine drug resistance, whether inherent or acquired, occurs in 30% of all ER-positive breast cancer patients [13], [14]. We reasoned that pertubation of molecular gene expression patterns in cells could lead to anti-endocrine resistance and that identification of these gene expression alterations could potentially lead to identification of novel and more effective therapeutic markers and targets.

In this report, we describe the construction of a large ∼400,000 member ZF-TF library and the identification of six library members capable of inducing stable, long-term anti-endocrine drug-resistance in breast cancer cells. High-density microarray analysis of differential gene expression patterns induced by these six artificial transcriptional factors revealed a common set of 72 target genes (a common fulvestrant-resistant gene expression signature) predicted to be involved in cellular pathways influenced by different classes of anti-endocrine agents. Highlighting the potential translational relevance of this approach, interrogation of the common fulvestrant-resistant gene expression signature in publicly available gene data sets demonstrated a positive association with poor prognosis in breast cancer patients. Taken together, our results demonstrate that artificial zinc finger transcription libraries can be used to induce stable drug-resistance in human cancer cell lines and to identify clinically relevant genes associated with the resistance phenotype.

## Results

### Construction of a combinatorial ZF-TF Library

**Figure 1:**
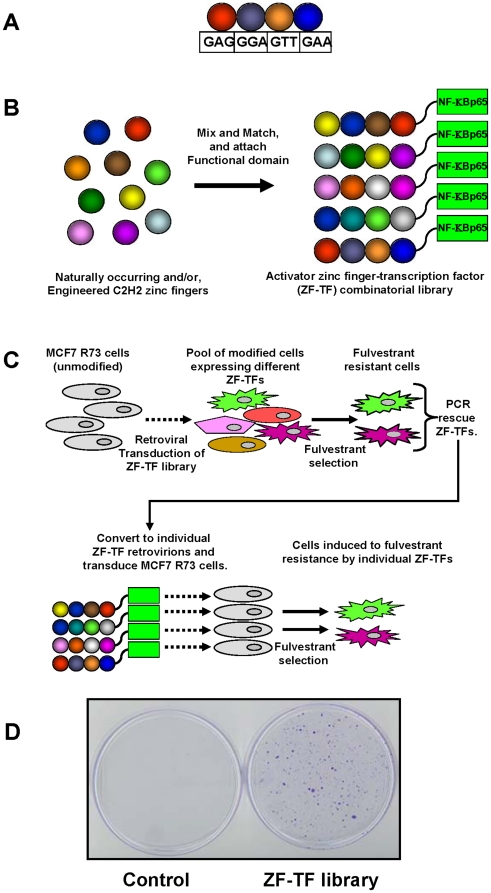
Schematic representation of ZF-TF combinatorial library screening approach.

Individual zinc finger domains typically bind to approximately 3 bp of DNA [4], [15]. These domains can be linked together into longer arrays of three or more fingers capable of recognizing longer DNA sequences (Figure 1A) [16], [17], [18]. Previous reports have described collections of various naturally occurring and engineered zinc finger domains with specificities for various three bp DNA sites. Several groups have created artificial ZF-TF libraries by randomly assembling combinations of individual zinc-finger domains with pre-characterized DNA-binding specificities into libraries of either three, four, or six-finger proteins [1], [2]. Each member of such a library has the potential to alter the expression of a spectrum of different genes in a cell, particularly if fused to a transcriptional regulatory domain (e.g.—an activation or a repression domain) [1], [2] Using 25 different zinc-finger domains (Table 1), we used a “mix and match” approach to create a combinatorial ZF-TF library consisting of as many as 390,625 (25^4^) different four-finger proteins each fused to a NF -KB p65 activation domain encoded in a retroviral vector that can confer puromycin-resistance (Figure 1B).

**Table 1:** Twenty-five individual zinc fingers used to construct the combinatorial ZF-TF library.

Zinc Finger Name	Expected Target Subsite (5′ to 3′)	Zinc Finger Amino Acid Sequence	Reference
LZF01	GAV	YKCKQCGKAFGCPSNLRRHGRTH	Bae et al., 2003 [19]
LZF02	GAW	YRCKYCDRSFSISSNLQRHVRNIH	Bae et al., 2003 [19]
LZF03	GGA	YKCGQCGKFYSQVSHLTRHQKIH	Bae et al., 2003 [19]
LZF04	HGA	YKCEECGKAFRQSSHLTTHKIIH	Bae et al., 2003 [19]
LZF05	GAA	FECKDCGKAFIQKSNLIRHQRTH	Bae et al., 2003 [19]
LZF06	NAA	YVCSKCGKAFTQSSNLTVHQKIH	Bae et al., 2003 [19]
LZF07	GYA	YKCPDCGKSFSQSSSLIRHQRTH	Bae et al., 2003 [19]
LZF08	HGA	YECHDCGKSFRQSTHLTQHRRIH	Bae et al., 2003 [19]
LZF09	GHG	YVCDVEGCTWKFARSDELNRHKKRH	Bae et al., 2003 [19]
LZF10	NGG	FQCKTCQRKFSRSDHLKTHTRTH	Bae et al., 2003 [19]
LZF11	GGG	YKCMECGKAFNRRSHLTRHQRIH	Bae et al., 2003 [19]
LZF12	GAG	YICRKCGRGFSRKSNLIRHQRTH	Bae et al., 2003 [19]
LZF13	AAT	YECDHCGKAFSVSSNLNVHRRIH	Bae et al., 2003 [19]
LZF14	GTD, GCD	YTCKQCGKAFSVSSSLRRHETTH	Bae et al., 2003 [19]
LZF15	GCW	YECNYCGKTFSVSSTLIRHQRIH	Bae et al., 2003 [19]
LZF16	GTG	FACPECPKRFMRSDALTRHIKTH	Liu et al., 2002 [18]
LZF17	GGC	FMCTWSYCGKRFTDRSHLARHKRTH	Liu et al., 2002 [18]
LZF18	GCC	FACPECPKRFMDRSDLTRHIKTH	Liu et al., 2002 [18]
LZF19	AAG	FACPECPKRFMRSDNLTQHIKTH	Liu et al., 2002 [18]
LZF20	RTC	YSCGICGKSFSDSSAKRRHCILH	Bae et al., 2003 [19]
LZF21	GRA, MGA	YVCRECGRGFRQHSHLVRHKRTH	Bae et al., 2003 [19]
LZF22	MAA	YMCSECGRGFSQKSNLIIHQRTH	Bae et al., 2003 [19]
LZF23	VGA, GAA	YECHDCGKSFRQSTHLTRHRRIH	Bae et al., 2003 [19]
LZF24	GGW	YRCEECGKAFRWPSNLTRHKRIH	Bae et al., 2003 [19]
LZF25	NNN	YACPVESCDRRFSRKWLLRLHIRIH	Joung et al., 2000 [17]

The full amino acid sequence of each finger and the expected target subsite(s) bound by each finger are shown.

### Identification of ZF-TFs that induced Fulvestrant-Resistance in MCF7 Cells

We used the approach outlined in Figure 1C to identify members of the ZF-TF library that were capable of inducing resistance to fulvestrant in breast cancer cells. For these experiments, we transduced MCF7-R73 cells, a monoclonal MCF7 subline that is highly sensitive to fulvestrant-induced cytocidal activity [19], with the ZF-TF activator library or with control retrovirus encoding only the NF-KB p65 activation domain. Both populations of cells were initially enriched for transduced cells by selecting for growth in puromycin and then for fulvestrant-resistant cells by selecting in the presence of 100 nM fulvestrant. After six weeks of continuous treatment with fulvestrant, hundreds of drug-resistant colonies emerged from the population of cells infected with the ZF-TF activator library (Figure 1D). By contrast, as expected, the control MCF7cells transduced by the control NF-KB p65-only retrovirus underwent massive cell death and therefore did not form drug-resistant colonies.

**Figure 2:**
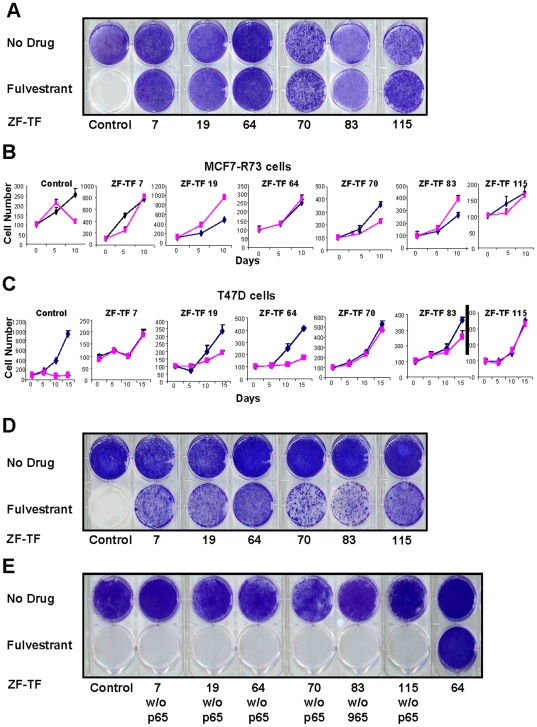
Fulvestrant resistance induced by 6 different ZF-TFs. (A) Drug sensitivity of fulvestrant-selected MCF7 ZF-TF-transduced cells. MCF7 cells transduced with one of six different ZF-TF-expressing retroviruses selected first in puromycin (the transduction selection marker) and then in fulvestrant for 1 month were grown in the absence of fulvestrant for 7 days and then challenged with 100 nM fulvestrant or vehicle (0.1% ethanol) for 21 days followed by crystal violet staining and visualization. Data are representative of triplicate experiments. (B and C) Growth curves of MCF7 and T47D cells in the presence and absence of fulvestrant. Comparison of cell growth rates (cell number, mean +/− SEM, n = 8; time in days as indicated) of MCF7 and T47D cells stably transduced with control retrovirus or one of six different ZF-TF-expressing retroviruses (7, 19, 64, 70, 83 and 115) in the presence (blue line) or absence (pink line) of fulvestrant. (D) Drug sensitivity of fulvestrant non-selected MCF7 ZF-TF cells. MCF7 cells transduced with one of six different ZF-TF-expressing retroviruses and selected in puromycin for 1 month were grown in the absence of fulvestrant for 7 days and then challenged with 100 nM fulvestrant or vehicle (0.1% ethanol) for 21 days followed by crystal violet staining and visualization. (E) Drug sensitivity of MCF cells transduced with ZF-TFs lacking the NF-KB p65 activation domain. MCF7 cells infected with retroviruses encoding ZF-TFs (7,19, 64, 70, 83 and 115) lacking the NF-KB p65 activation domain were selected in puromycin for 1 month and then challenged with 100 nM fulvestrant or vehicle (0.1% ethanol) for 21 days followed by crystal violet staining for visualization.

DNA encoding ZF-TFs was rescued by PCR from the genomic DNA of pooled fulvestrant-resistant cells. The sequences of the individual ZF-TFs were determined and 46 unique ZF-TF clones identified. These 46 unique ZF-TFs were re-cloned into the retroviral vector and converted into clonal virus stocks that were used to transduce MCF7-R73 cells. These 46 retrovirally transduced cell populations were then challenged with fulvestrant (Figure 1C). Compared with the control MCF7-R73 cells transduced with the NF-KB p65-only retrovirus (hereafter referred to as MCF-238 cells), the MCF7-R73 cells transduced with six of the 46 unique ZF-TFs demonstrated survival and growth in the presence of 100 nM fulvestrant (Figures 2A and B). The sequences of the six ZF-TF arrays conferring fulvestrant resistance are presented in Table 2. To test whether these six ZF-TFs could induce fulvestrant-resistant in cells other than MCF7-R73, T47D breast cancer cells, a second fulvestrant-sensitive ER+ human breast cancer line, were individually transduced with each of the six different ZF-TFs and challenged with fulvestrant. Similar to what was observed with MCF7-R73 cells, the six ZF-TFs conferred resistance to fulvestrant-induced growth inhibition in T47D cells (Figure 2C). Consistent with its reported mechanism of action, fulvestrant suppressed ER−alpha expression in all ZF-TF-induced resistant sublines and the sensitive MCF7-238 control subline (Figure S1). Given that fulvestrant suppressed ER− alpha expression to a level equal to or greater than that observed in the control cells, it is unlikely that drug resistance was caused by enhanced drug metabolism or active drug exclusion. In order to assess the latter possibility, we performed drug sensitivity testing as previously described [20] to 24 chemotherapeutic agents and investigational compounds (Table S1) in three different ZF-TF-transduced fulvestrant-resistant cell lines. Comparison of the control MCF7-R73 cells to the three ZF-TF fulvestrant resistant cell lines revealed no significant difference in the pattern of drug resistance and sensitivity suggesting that the ZF-TFs are not inducing a multi-drug resistance phenotype (Figure S2).

**Table 2:** Composition of Zinc Finger-Transcription Factor Arrays Conferring Fulvestrant Resistance.

ZF-TF clone #	Finger 1	Finger 2	Finger 3	Finger 4
7	LZF15	LZF23	LZF10	LZF20
19	LZF10	LZF02	LZF18	LZF12
64	LZF08	LZF25	LZF11	LZF23
70	LZF12	LZF03	LZF04	LZF21
83	LZF10	LZF25	LZF19	LZF23
115	LZF13	LZF14	LZF13	LZF23

Full details and sequences of the individual fingers shown for each ZF-TF are provided in Table 1.

To rule out that selection in fulvestrant was required for the phenotype of ZF-induced drug resistance, we transduced MCF7-R73 cells with the six ZF-TFs, subjected the cells to 3 weeks of puromycin selection, and then challenged the transduced cells with 100 nM fulvestrant. Despite the absence of selection in fulvestrant, all six ZF-TFs again conferred fulvestrant resistance (Figure 2D). To determine if the NF-KB p65 activation domain is required for ZF-TF-induced fulvestrant resistance, MCF7-R73 cells were transduced with retrovirus expressing only the ZF-TFs (i.e.-without a fused p65 activation domain) and challenged with fulvestrant. These transduced MCF7-R73 cells were all found to be sensitive to fulvestrant-induced cell growth inhibition and cell death (Figure 2E), demonstrating that the NF-KB p65 activation domain is required for ZF-TF-induced fulvestrant resistance.

### Gene Expression Profiling

**Figure 3:**
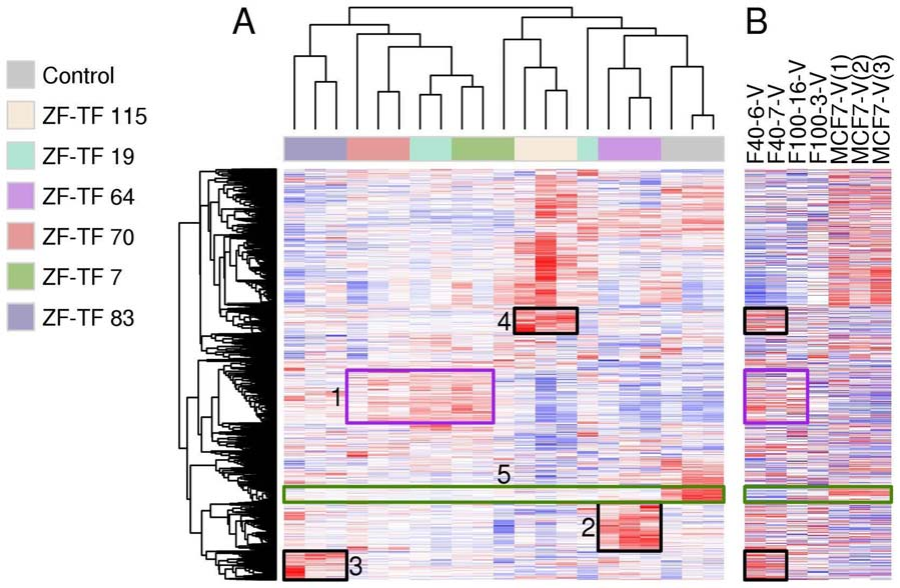
Clustering of expression profiles from ZF-TF-induced fulvestrant resistant cells. (A) MCF7 cells transduced with ZF-TF-encoding retrovirus or a control virus. Green box identifies a cluster of 72 genes that are consistently down-regulated by ZF-TF expression- these genes constitute the common fulvestrant-resistant gene expression signature. Black and purple boxes illustrate that gene clusters that are up-regulated by subsets of the six different ZF-TFs. Data represent gene expression profiles from replicate retroviral infections and microarray hybridizations. (B) Pre-existing fulvestrant resistant clones and control MCF-7 cells from Coser et al [19]. Genes are arranged in the same order as in (A). Green box shows that genes down-regulated by all the ZF-TF infections were also down-regulated in the pre-existing fulvestrant resistant sub-population. Black and purple boxes show that some of the gene clusters that were up-regulated by various ZF-TFs were also up-regulated in either two or three (F40-6-V, F40-7-V and F100-16-V) of the four previously described pre-existing fulvestrant resistant subpopulations in the MCF7 cell line [19].

To determine the transcriptional alterations associated with ZF-TF-induced fulvestrant resistance, we performed comparative gene expression profiling of each of the six ZF-TF-transduced MCF7 cell lines relative to the control MCF7-238 cells. Fulvestrant exposure induces massive cell death in our control MCF7-238 cells. Thus, in order to avoid the confounding effects of cell death-associated gene expression alterations, we performed our expression profiling analysis using MCF7-238 cells and ZF-TF-induced fulvestrant resistant cells grown in the absence of fulvestrant for 3 days. Cluster analysis of the differential expression profiles of the ZF-TF-infected cells and the control cells identified a shared cluster of genes up-regulated by ZF-TF 7, 19 and 70 (Figure 3A, purple rectangle, henceforth gene cluster 1), and distinctive gene expression patterns induced by ZF-TF 64, 83 and 115 (Figure 3A, black rectangles, henceforth gene clusters 2, 3 and 4, respectively). A gene cluster consistently up-regulated in all six fulvestrant resistant cell lines was not identified. However, a 72-gene cluster was consistently down-regulated in all six fulvestrant resistant cell lines (Figure 3A, green rectangle, henceforth gene cluster 5), and this cluster constituted a common fulvestrant-resistant gene expression signature (Table S2).

To gain additional insights into the biological processes in which these various differentially expressed genes are involved, we performed hypergeometric gene set enrichment analysis (GSEA) of the different ZF-TF-induced gene set clusters using the BROAD Institute MSigDB gene set database augmented with ESR1, ERBB2 and proliferation coexpression modules [21], [22], MYB regulated genes [23] and a signature of long-term estrogen deprivation [24]. Two prominent findings emerged from this analysis. First, the shared cluster of up-regulated gene expression induced by ZF-TF 7, 19 and 70 (cluster 1) and the unique gene clusters (cluster 2, 3 and 4) induced by ZF-TF 64, 83 and 115, respectively, show significant overlap with myb-regulated genes (Tables S3, S4, S5, S6). Second, and most notable, gene set enrichment analysis of the common fulvestrant resistant gene expression signature (cluster 5) revealed significant overlap with gene sets associated with estrogen receptor negative human breast cancers, with estradiol responsiveness, and with anti-endocrine drug resistance to tamoxifen [25], [26], [27], fulvestrant [26] and aromatase inhibitors [25] in multiple breast cancer cell lines including long-term estrogen-deprived (LTED) cells [24]. (Table S7). Thus, the common fulvestrant-resistance gene expression signature induced by six different ZF-TFs represents an estrogen receptor-negative-like transcriptional state that is associated with an anti-endocrine resistance phenotype.

We next sought to compare the patterns of gene expression observed in our ZF-TF-induced fulvestrant resistant MCF7 cells with those found in fulvestrant-resistant MCF-7 progenitor cells previously identified by another group [19]. These fulvestrant-resistant clones isolated by Coser et al. represent rare, pre-existing cells from the heterogeneous mixture of cells found in the MCF7 cell line [19]. Several of the gene clusters (clusters 1, 3 and 4) that were up-regulated by the ZF-TFs were also selectively up-regulated in some of the naturally selected pre-existing fulvestrant resistant cells [19] (Figure 3 B, black and purple rectangles). ZF-TF 64 was associated with a unique set of upregulated genes (cluster 2), not observed by Coser et al. in any of their previously published fulvestrant-resistant clones. However, the common fulvestrant-resistant gene expression signature (cluster 5) was also observed in the fulvestrant-resistant cells identified by Coser et al. (Fig. 3B, green rectangle). Thus, taken together, these results confirm previous findings by Coser et al. and, also provide evidence for the existence of alternative molecular pathways associated with fulvestrant resistance.

**Figure 4:**
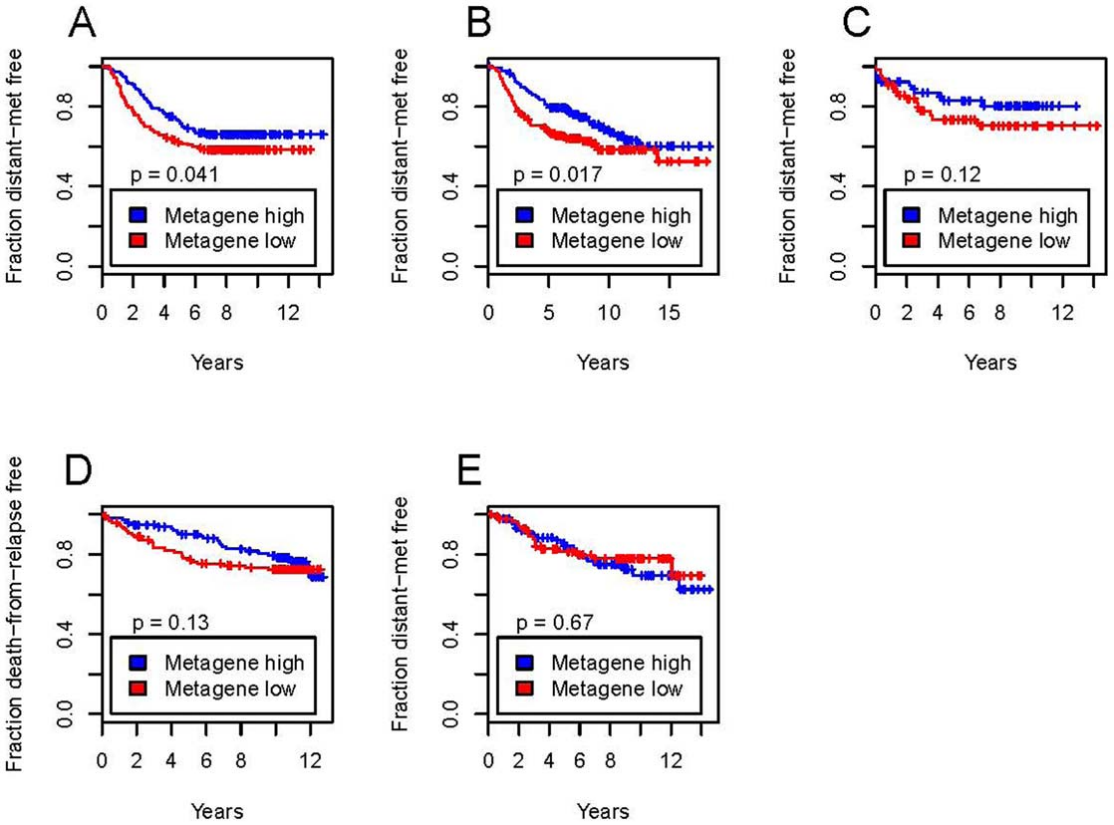
Prediction of breast cancer outcome using a fulvestrant-resistant gene signature identified by our ZF-TF-based approach. Patients were stratified using a metagene that was formed by averaging the expression of the 72 genes constituting the common signature for the fulvestrant resistance phenotype (i.e., the genes of cluster 5 in Fig. 3A). Tumors with metagene values below the median were defined as the “fulvestrant-resistant metagene” and the others as the “fulvestrant-sensitive metagene.” Kaplan-Meier curves for distant metastasis-free survival or death-from-relapse-free survival using the metagene are based on clinical data described by (A) Wang et al., N = 286 [28], (B) van de Vijver et al., N = 295 [29], (C) Chin et al., N = 130 [30], (D) Miller et al., N = 251 [31] and (E) Sotiriou et al., N = 189 [32]. p-values are one-sided.

To determine the potential clinical relevance of our approach, we assessed whether the common fulvestrant-resistant gene expression signature (cluster 5) might have prognostic value for patients with breast cancer. We hypothesized that a drug resistance phenotype would be associated with a more aggressive clinical course and thus its gene expression signature would correlate with poor prognosis. To test this hypothesis, we formed a “fulvestrant-resistant metagene” signature by averaging the expression of the 72 genes (cluster 5) that constitute the common fulvestrant resistance gene expression signature, and tested this metagene in five independent previously published human breast cancer data sets by Wang et al. (286 patients) [28], van de Vijver et al. (295 patients) [29], Chin et al. (130 patients) [30], Miller et al. (251 patients) [31] and Sotiriou et al. (189 patients) [32]. In four of five data sets, fulvestrant resistant-metagene values (i.e. tumors that express the fulvestrant resistance gene expression signature) correlated with poor prognosis, significantly so in two of the data sets (Figure 4). These clinical correlation data support the notion that our ZF-TF-based approach can be used to develop biomarkers with potential clinical relevance.

## Discussion

Previous advances in the clinical treatment of breast cancer have been strongly influenced by data from *in vitro*-based approaches in which drug-resistant cells emerge under selective drug pressure [33], [34], [35]. In this study, we undertook a different approach in which we identified six members of a combinatorial zinc finger transcription factor library capable of inducing stable anti-endocrine drug resistance in a clonal breast cancer cell line. Our results stand in contrast to other recent studies in which *in vitro*-based drug resistance in breast cancer cells have arisen by the selection of pre-existing drug-resistant progenitor cells [19]. Differential gene expression analysis identified a common set of genes that were all down-regulated by the six artificial ZF-TFs. Because our ZF-TFs are expected to be activators of gene expression, we hypothesize that repression of these common genes is indirect, presumably through activation of one or more transcriptional repressor proteins.

Interestingly, gene set enrichment analysis of the common fulvestrant resistance gene expression signature (cluster 5) revealed that it is composed of genes whose expression is modulated by estradiol [27], [36], [37], [38]. Because all of these genes were decreased in their expression, the ZF-TF-induced fulvestrant resistance we observed is likely to be associated with the generation of an “estrogen receptor-negative” cell state. These findings are consistent with that of Creighton et al. in which tamoxifen-resistant and fulvestrant resistant MCF7-HER2-overexpressing xenografts shut down classic estrogen receptor gene expression signaling pathways [26]. Together these observations are reminiscent of the clinical setting in which anti-estrogen resistant human breast cancers behave in an aggressive estrogen-independent manner [14]. In addition, our GSEA results confirmed the relevance our ZF-TF approach, as our common fulvestrant resistance gene expression signature demonstrated highly significant overlap with multiple previously described breast cancer anti-hormonal resistance gene sets. Specifically our signature demonstrated significant overlap with those observed for tamoxifen, fulvestrant and aromatase inhibitor resistance in multiple breast cancer cell lines including long term estrogen deprived (LTED) cells [24], [25], [26], [27] . Whether any single genes or subset of genes within these overlapping gene sets serves as a “passenger” or “driver” of anti-hormonal resistance remains to be determined.

In addition, our gene set enrichment analysis also identified four unique *myb*-regulated gene sets in the ZF-TF-induced fulvestrant resistant cells. Intriguingly, c-myb is expressed in a high proportion of ER-positive breast cancers [39] and plays a pro-proliferative role in ER-positive, but not ER-negative breast cancer [40]. Expression of c-myb is regulated by estrogen and antiestrogens, and is altered in long-term estrogen deprived (LTED) breast cancer cell line model of estrogen-independent growth [41]. Our analyses identified four unique and independent myb-regulated gene sets, rather than a single overlapping set, a result similar to that observed by Lei et al. [23]. More specifically, expression of myb proteins in different cell types resulted in the activation of unique and nearly non-overlapping sets of genes in each cellular context. Furthermore, deletion and domain swap experiments resulted in the identification of unique positive and negative elements in myb that regulate different cassettes of gene expression [23]. Thus, we hypothesize that the mostly non-overlapping sets of myb-regulated genes are up-regulated by the different ZF-TFs in various contexts. Furthermore, taken together, our data suggest that c-myb's potential role in anti-estrogen resistance extends beyond the mere expression of c-myb itself and is likely influenced by other as yet to be defined factors, which may serve as interesting avenues for future research.

Our results provide another important proof-of-principle that ZF-TF library screening can be used to identify genes associated with specific phenotypes of interest. One of the strengths of the ZF-TF approach is that each member of the library has the potential to regulate the expression of dozens to hundreds of genes in a cell. This promiscuity, seen in our gene expression analysis, is not unexpected given the frequency with which any 9 to 12 bp site (the length of DNA that will likely be bound by a four-finger protein) will occur just by chance in random sequence and stands in contrast to other RNAi or cDNA overexpression libraries where individual members of the library are expected to be specific in their effects. We speculate that this ability of a single ZF-TF to regulate multiple genes might provide an advantage for inducing complex phenotypes that may require the alteration of multiple cellular pathways. Earlier studies have suggested that differential gene expression analysis of multiple ZF-TFs can help to define genes with altered expression associated with an induced phenotype [7]. Our study includes the largest number of ZF-TFs that have been profiled for a single induced phenotype and lend important additional support to the concept that genes responsible for phenotypes can be deduced despite the relative promiscuity of the individual ZF-TFs.

Although the combinatorial ZF-TF library we constructed for this report consists of zinc finger arrays linked to a transcriptional activation domain, additional libraries consisting of fusions to other functional domains could also be easily constructed. Building of such libraries can be easily performed because our library is encoded on a plasmid in which the coding sequences of the zinc fingers are flanked by Gateway recombination sites. As we did for the experiments of this study, the zinc finger library coding sequences can therefore be readily transferred to any appropriate Gateway Destination vector and could be easily fused to various other functional domains (e.g.—transcriptional repression domains, histone modification domains, or DNA methyltransferases). We also note that the Gateway cloning sites in our library vectors greatly simplified rescue of zinc finger coding sequences from genomic DNA of drug-resistant cells and subsequent regeneration of viral vectors.

In summary, our results provide another important proof-of-principle for the use of combinatorial ZF-TF libraries to induce and to study important cellular phenotypes. The features of our combinatorial library should facilitate construction of additional ZF fusion protein libraries and simplify the rescue of ZF fusions from cells exhibiting the desired phenotype. Because members of the combinatorial library are predicted to have diverse effects on a cell and because zinc fingers are known to bind DNA in cell types ranging from humans to bacteria, we envision that this broadly applicable tool will play an important role in functional genomics and bioengineering for a wide variety of different organisms. The results from this study underscore the clinical relevance of our approach and serve as a relevant model for studying cancer drug resistance.

## Materials and Methods

### Cell Culture and Reagents

MCF7-R73 cells, a clonal isolate derived from the MCF7 cell line were kindly provided by Dr. Toshi Shioda (MGH, Charlestown, MA) and maintained in a xenoestrogen controlled environment as described [19], and the T47D and 293T cells were obtained from American Type Culture Collection (Manassas, VA) and maintained as described. Fulvestrant was purchased from Selleck Chemicals LLC (Houston, TX). Puromycin was purchased from Sigma-Aldrich (St.Louis, MO).

### ZF-TF Retroviral Library Production and Drug Resistance Screening

293T cells were seeded at 5×10^5^ cells per 10 cm culture dish, transfected with 3.2 micrograms of ZT-TF p65 activator library DNA and 2.4 micrograms and 0.8 micrograms of PMD-MLV and PMD-G plasmid DNA using Fugene 6 (Roche Applied Science, Indianapolis IN) according to the manufacture's protocol and retroviral supernatant harvested 48 hrs later. MCF7-R73 cells (6×10^5^ per 10 cm plate) were infected with 1.33×10^5^ ZF-TF retrovirions in media containing polybrene. Two days post-infection the cells were exposed to puromycin (0.4 mg/ml), and three days later were subjected to continuous combined puromycin and fulvestrant (100 nM) selection for an additional 42 days. Retroviral DNA was recovered by PCR, subcloned into the pDONOR plasmid using Gateway technology (Invitrogen), and subject to DNA sequencing that revealed 46 unique ZF-TF arrays. The 46 ZF-TFs were re-cloned into the retroviral destination plasmid using Gateway technology, 46 ZF-TF retroviral supernatants generated, and MCF-R73 cells infected and subjected to puromycin and fulvestrant selection. Six unique ZF-TFs conferred drug resistance in the presence of continuous fulvestrant exposure. A control MCF7 cell line (MCF7-R238) was generated by infecting MCF7-R73 cells with a retrovirus bearing the p65 activation domain only. T47D cells were infected with the six unique ZF-TF bearing retroviruses and subjected to fulvestrant exposure as described for the MCF7 cells.

### Growth Assays and Crystal Violet Staining

ZF-TF infected MCF7 and T47D cells, and corresponding control cells were seeded at 1 or 2×10^3^ cells per 96-well plate and grown at 37°C in a humidified incubator containing 5% CO_2_ in the presence of puromycin (0.4 micrograms/ml) only and puromycin plus 100 nM fulvestrant for 10–15 days. At the designated time points, cells were washed by PBS, fixed with 4% formaldehyde in PBS for 20 minutes, washed four times with distilled water, and stained with the Syto 60 (Invitrogen) nuclear stain for 45 minutes in a dark room. Syto 60 staining was measured with an Odyssey (LI-COR) 96-well plate reader, and values normalized as % control at 0 day point in puromycin (0.4 micrograms/ml) only treated cells and puromycin plus 100 nM fulvestrant treated cells. The results were expressed as means + standard errors for 8 replicate determinations for each experiment. For crystal violet staining assays 1×10^5^ cells were seeded in each well of 6 well plate, treated with puromycin (0.4 micrograms/ml) only or puromycin in combination with 100 nM fulvestrant for 3 weeks (or no fulvestrant was treated for 3 weeks, washed in PBS, fixed with 4% formaldehyde in PBS, and stained with 0.2% crystal violet in 10% ethanol. Stained cells were washed multiple times with distilled water, dried and images captured.

### Gene Expression Profiling

Transcriptional profiling was performed using Affymetrix U133-plus 2.0 microarrays as previously described [42]. Control and ZF-TF-infected MCF7 cells were profiled from two independent infections and the profiling was performed in four batches. The MAS5 algorithm was used to produce non-log-transformed expression values such that the 2% trimmed mean of the expression values for each sample was 100. The expression values were corrected for batch effect on a per-probe-set basis as follows. For each batch N other than the first, we added to the expression values of batch N the difference between the average expression value on the first batch and the average expression value on batch N. All microarray data are MIAME compliant and the raw data has been deposited in the NCBI Gene Expression Omnibus and are accessible [GEO: GSE27444].

### Clustering Analysis

The probe-sets with the top 2,500 standard deviations in expression value were retained and the others discarded. The resulting matrix was median polished and then each row was scaled to have mean 0 and standard deviation 1. The resulting matrix was clustered using average-linkage agglomerative hierarchical clustering with distance given by one minus the correlation coefficient. From the pre-existing fulvestrant resistant sub-population and control data the same 2,500 probe-sets were retained and the rest discarded. The resulting matrix was median polished and then each row was scaled to have mean 0 and standard deviation 1.

### Hypergeometric Gene Set Enrichment Analysis

A gene-set database was formed by augmenting version 3.0 of the Broad Institute's MSigDB gene set database [21] with the six gene sets found by Wirapati, et. al. [22], to be either correlated or anti-correlated with ESR1, ERBB2 and AURKA, and with the myb-regulated genes defined in the supplementary data of Lei et al. [23] and with the 99 gene LTED signature defined in supplementary table S4 of Miller et al. [24]. For each gene cluster in Fig. 3A, we did the following. For each gene-set in the gene-set data base, a hypergeometric test was performed to determine whether the intersection of the gene-set with the genes in the gene cluster in Fig. 3A was larger than would be expected by chance had both been drawn at random from all the genes on the Affymetrix U133-plus 2.0 microarray. The resulting p-values were adjusted by the Holm method to control family-wise error rate (FWER).

### Kaplan-Meier Analysis

A metagene was formed by averaging the expression of the 72 genes that constituted a common signature for the fulvestrant resistance phenotype (i.e., the genes of cluster 5 in Fig. 3A). Tumors with metagene values below the median were defined to be “fulvestrant- resistant” and the others “fulvestrant sensitive.” Log-rank test was used to test the hypothesis that fulvestrant-resistant metagene tumors would have worse survival than fulvestrant-sensitive metagene tumors. If distant metastasis-free survival data was available for a particular data set, it was utilized; otherwise death-from-relapse-free survival data was used. P-values are one-sided.

## Supporting Information

Figure S1 **Expression of ER−alpha in the ZF-TF-transduced fulvestrant resistant cells.** Control-transduced (R238) and ZF-TF transduced fulvestrant resistant cells (F7, 19, 64, 70, 83 and 115) were exposed to vehicle or fulvestrant (100 nM) for 6 days. Cells were lysed in RIPA buffer containing protease inhibitor, and 100 micrograms of each lysate was loaded onto a 10% SDS-PAGE gel and subjected to Western blotting as previously described [43] using ER-alpha and beta-actin antibodies (Santa Cruz).
(TIF)

Figure S2 **Drug Sensitivity of ZF-TF-transduced fulvestrant resistant cells.** Control MCF7 (R238 cells) and ZF-TF fulvestrant resistant (F7, 19, 64, 70, 83 and 115) cells (5×103) were exposed to vehicle or 20 uM of multiple selective target inhibitors (see supplemental Table 1) for 72 hrs as previously described. Cell growth/viability was quantitated by fluorescent nucleic acid staining of fixed cells using Syto60 (Molecular probes) and a SpectraMax M5 plate reader. Each bar represents signal intensity of the treated cells relative to the non-treated cells, and drug sensitivity is calculated as the fraction of viable treated-cells relative to untreated-cells within each cell type. A single and double asterisk represents significant (p<0.05) drug sensitivity in which the value of signal intensity of treated cells relative to that of non-treated cells is 0.5–0.75 and 0.2–0.5, respectively; pink-colored bar represents similar drug sensitivity pattern to the parental cell line, R73. The frequency of values falling in each group from different cell lines were tested against the frequency of values expected in each group from the same cell lines by Chi Square analysis; differences in drug sensitivity between the control cells (R73) and the ZF-TF transduced cells (F11, F19 and F64) were not statistically significant.
(TIF)

Table S1 **Chemotherapeutic agents and investigational compounds used in the drug sensitivity screen.**
(XLS)

Table S2 **Genes (72) constituting the common fulvestrant-resistant gene expression signature.**
(XLS)

Table S3 **Gene Sets enriched in ZF-TF 7, 19, and 70-induced fulvestrant resistant cells (Gene Cluster 1: 230 genes).**
(XLS)

Table S4 **Gene Sets enriched in ZF-TF 64-induced fulvestrant resistant cells (Gene Cluster 2: 225 genes).**
(XLS)

Table S5 **Gene Sets enriched in ZF-TF 83-induced fulvestrant resistant cells (Gene Cluster 3: 135 genes).**
(XLS)

Table S6 **Gene Sets enriched in ZF-TF 115-induced fulvestrant resistant cells (Gene Cluster 4: 123 genes).**
(XLS)

Table S7 **Gene Sets enriched in all ZF-TF-induced fulvestrant resistant cells (Gene Cluster 5: 72 genes).**
(XLS)
