# Supplementary figures and images for: Induction of Stable Drug Resistance in Human Breast Cancer Cells Using a Combinatorial Zinc Finger Transcription Factor Library
Source: PLoS One. 2011 Jul 19;6(7):e21112. doi: 10.1371/journal.pone.0021112 (PMC3139592; doi:10.1371/journal.pone.0021112)

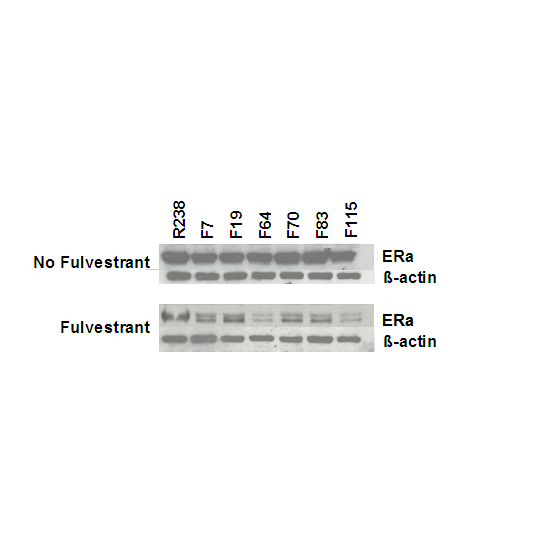

Supplement: Figure S1 — Expression of ER−alpha in the ZF-TF-transduced fulvestrant resistant cells. Control-transduced (R238) and ZF-TF transduced fulvestrant resistant cells (F7, 19, 64, 70, 83 and 115) were exposed to vehicle or fulvestrant (100 nM) for 6 days. Cells were lysed in RIPA buffer containing protease inhibitor, and 100 micrograms of each lysate was loaded onto a 10% SDS-PAGE gel and subjected to Western blotting as previously described [43] using ER-alpha and beta-actin antibodies (Santa Cruz). (TIF) [file pone.0021112.s001.tif]

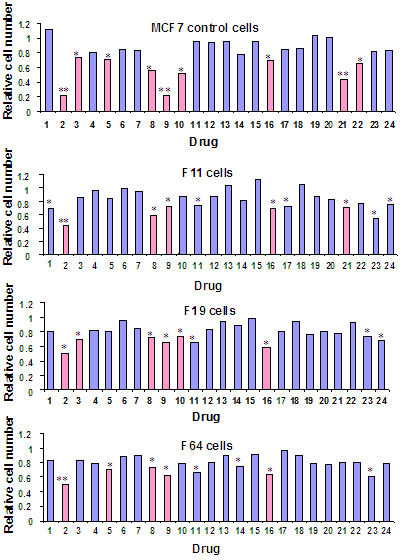

Supplement: Figure S2 — Drug Sensitivity of ZF-TF-transduced fulvestrant resistant cells. Control MCF7 (R238 cells) and ZF-TF fulvestrant resistant (F7, 19, 64, 70, 83 and 115) cells (5×10^3^) were exposed to vehicle or 20 uM of multiple selective target inhibitors (see supplemental Table 1) for 72 hrs as previously described. Cell growth/viability was quantitated by fluorescent nucleic acid staining of fixed cells using Syto60 (Molecular probes) and a SpectraMax M5 plate reader. Each bar represents signal intensity of the treated cells relative to the non-treated cells, and drug sensitivity is calculated as the fraction of viable treated-cells relative to untreated-cells within each cell type. A single and double asterisk represents significant (p<0.05) drug sensitivity in which the value of signal intensity of treated cells relative to that of non-treated cells is 0.5–0.75 and 0.2–0.5, respectively; pink-colored bar represents similar drug sensitivity pattern to the parental cell line, R73. The frequency of values falling in each group from different cell lines were tested against the frequency of values expected in each group from the same cell lines by Chi Square analysis; differences in drug sensitivity between the control cells (R73) and the ZF-TF transduced cells (F11, F19 and F64) were not statistically significant. (TIF) [file pone.0021112.s002.tif]
